# Comparative analysis of visit and home blood pressure in a pilot trial on the effect of 18% sodium substitute salt on blood pressure

**DOI:** 10.1038/s41598-020-79282-2

**Published:** 2021-01-13

**Authors:** Ting Liu, Huakun Rao, Meixian Wang, Huini Xu, Wen Wang, Ge Li, Hao Wang, Lihong Mu

**Affiliations:** 1 Department of Epidemiology, School of Public Health and Management, Research Center for Medicine and Social Development, Innovation Center for Social Risk Governance in Health, Chongqing Medical University, Chongqing, 400016 China; 2 Chongqing Nan’an District People’s Hospital, Chongqing, China

**Keywords:** Disease prevention, Health services, Nutrition, Public health

## Abstract

Aim to compare the home blood pressure monitoring (HBPM) and visit blood pressure monitoring in a clinical phase I single-arm pilot trial. The 18% sodium substitute salt was used in 43 hypertensives for 8 weeks, and visited once a week, while weekly visit blood (VBP) pressure, daily home blood pressure (HBP) and urine test results before and after intervention were collected. 43 hypertensive patients were recruited, 4 were lost. And enrolled 39 patients for analysis. The VBP were lower than morning HBP and night HBP (*P* < 0.05). And VBP was good correlated with morning BP (SBP: r = 0.692, *P* < 0.001, DBP: r = 0.789, *P* < 0.001) and night BP (SBP: r = 0.571, *P* < 0.001, DBP: r = 0.738, *P* < 0.001). The results of mixed linear model analysis showed that patients' visit SBP (− 11.4 mmHg, 95% CI: − 17.0 to − 5.7, *P* < 0.001), morning home SBP (− 10.0 mmHg, 95% CI: − 16.4 to − 3.6, *P* = 0.003) and night home SBP (− 10.2 mmHg, 95% CI: − 15.8 to − 4.6, *P* = 0.001) decreased significantly, after intervention. Both HBP and VBP showed that 18% substitute salt intervention could decrease the blood pressure of hypertensives. Medication led to VBP lower than HBP, but the two still had a good correlation.

*Trial registration*: NCT03226327. Registered 21 July 2017—Retrospectively registered, http://www.clinicaltrials.gov.

## Introduction

As a leading risk factor for cardiovascular diseases^1,2^, hypertension has become one of the main burdens of human health, and excessive salt intake is closely related to an increase in blood pressure and the risk of cardiovascular-related events^3^. The World Health Organization recommends that the daily salt intake per person should be less than 5 g per day, while current salt intake is about 12 g or more in many countries, including China^4^. Therefore, it is imperative to find an effective salt restriction measure. With substitute salt as an effective means to reduce sodium intake, its antihypertensive effect has been widely recognized^5–9^. However, the sodium content of substitute salts in previous studies is generally high, mostly around 70%^7,8^, and some may be as low as 50% or even 25%^9,10^. And substitute salt of different ingredients may have different effects of lowering blood pressure. What’s more, in the case of relatively high sodium intake, some previous salt-restricted studies have also shown that the lower the sodium intake, the lower the blood pressure^11–13^. For the first time, this study applied substitute salt with only 18% sodium to patients with hypertension, and observed the effect of blood pressure reduction.

In addition, blood pressure as a key indicator, choosing a superior approach to measure it will be crucial. There are many studies on comparing different types of blood pressure monitoring^14–22^, but there are few comparative studies on two types of blood pressure monitoring in prospective intervention studies.

In the actual intervention studies, taking into account the cost of research and the convenience of implementation, usually, previous intervention studies used visit blood pressure monitoring (VBPM) to observe changes in blood pressure during the study period. In recent years, with the increasing emphasis on the home blood pressure (HBPM) and its popularity, more and more studies have adopted HBPM^23–25^. Then, what is the difference between the two kinds of blood pressure monitoring in the intervention studies? And what different blood pressure monitoring will bring to the research results?

## Method

### Participants

Hypertensive patients under the jurisdiction of a hospital in Chongqing Municipality, China, from May to July 2018. This study was designed as a Phase I clinical trial involving no less than 30 patients in accordance with international practice^26^.

Inclusion criteria: (1) aged ≥ 50 and ≤ 75 years; (2) not had plans to move out of the community in the next three months; (3) not cooking at home less than 3 times or one day during the study; and (4) provided written informed consent before enrollment in the trial. Exclusion criteria: (1) history of acute myocardial infarction or stroke in the past 3 months, history of malignancy or expected lifetime less than 1 year; (2) hypercortisolism or aldosteronism; (3) acute disease, such as upper respiratory infection, fever and diarrhea; (4) salt substitute use in the family; (5) disease or disabilities that could exert potential influence on their adherence to the intervention, including deafness, dementia, as well as severe depression and other mental disorders; (6) family members not willing to use the salt substitute; (7) liver dysfunction; (8) anyone with abnormal serum potassium in family; (9) anyone using potassium-retaining diuretics in family.

The Institutional Review Board of Peking University has approved the experiments, including any relevant details (IRB00001052-17110) which means that all experiments were performed in accordance with relevant guidelines and regulations.

### Study design and intervention

It’s based on a clinical phase I trial, after patients and their family signed the informed consent forms, subjects completed baseline questionnaires and medical examination. 18% substitute salt instead of traditional salt was used for 8 weeks, and patients measured home blood pressure every day, while visit staff visited once a week to measure the blood pressure of patients and collect the information of antihypertensive drugs use, dosages of sodium-limited formula salts, and adverse events, and 24-h urine was collected again at week 8 and sent for examination.

### Materials and instruments

In this study, 18% substitute salt of "Man Li Kang" was developed by Chongqing Institute of Biotechnology Co., Ltd. Name: solid compound condiment, standard of execution: Q/SWS 0025 S, food production license number: SC10650012000709, food circulation permit: SP5009051610016538, and the main ingredients include potassium chloride (35%), sodium chloride (18%), calcium chloride (10%). The blood pressure measuring instrument adopt validated automated upper-arm cuff devices that operate through the oscillometric technique (Manufacturer: Shenzhen Raycome Health Technology Co., Ltd. Product name: “Maibobo” electronic sphygmomanometer. Model: RBP-9801). The urine samples were sent to the First Affiliated Hospital of Chongqing Medical University laboratory, and the enzyme was detected by HITACHI R7600.

### Blood pressure measurement

Before the start of the study, visit staff were trained in blood pressure measurement related knowledge, followed by one-on-one blood pressure measurement training for each patient by the visit staff, and a home blood pressure measurement handbook was distributed to the patient.

The visit staff measured the patient's blood pressure once a week as a visit blood pressure, requiring three blood pressure measurements to be completed before 10:00 am, interval of about 2 min.

While, patients were required to complete 3 blood pressure measurements a time in the morning and evening (from 6 to 9 o'clock am/pm) every day. Besides, during the weekly visit, the visit staff will confirm with the patients whether the blood pressure measurement method is correct or not. And the blood pressure measurement results will be automatically uploaded to the terminal management system in real time through the devices.

### Data analysis

Each time, 3 blood pressure measurements were collected, and the last 2 averages were included in the analysis. And home blood pressure was converted to weekly blood pressure according to the European Hypertension League guidelines^27^.

Statistical analysis was carried out using SPSS 22.0 (IBM, Armonk, NY, USA). Visit blood pressure data is complete. And a total of 1953 pieces of complete home blood pressure measurements data were collected in the morning, the missing rate was 9.45%, and 1871 in the evening, with a missing rate of 14.33%. And the missing data of home blood pressure was filled by multiple imputation^28^. Quantitative normal distribution data was described by mean and standard deviation, and paired T test was used for comparing the difference before and after intervention. Qualitative data is described by frequency. Non-positive distribution data are expressed in median and quartiles, and Wilcoxon Signed Rank Test was used. Analysis of variance for comparing visit blood pressure and home blood pressure, Least-Significant Difference (LSD) for comparison between tow samples, and Pearson correlation analysis for analysis of blood pressure correlation. The linear mixed models were used to analyze the change of the blood pressure. *P* values less than 0.05 were considered statistically significant.

## Result

### Baseline

In the study, we screened 69 patients, 26 of whom were excluded because they did not meet the exclusion criteria (excluding criteria 2 and 6), and eventually included 43 patients, 4 lost because of the intolerability of the taste from the substitute salt.

Analyze by per protocol (PP), and incorporated 39 patients, including 20 males and 19 females, aged 67.1 ± 7.9 years old, BMI (Body Mass Index) was 26.6 ± 3.6. Among them, 5 patients currently have the habit of drinking, and drink ≤ 7 times a week, with an average alcohol consumption of ≤ 100 ml each time. The average age of hypertension was 11.0 ± 6.3 years. Blood pressure and medication at baseline are shown in Table 1.

**Table 1:** Sociodemographic and clinical characteristics.

Characteristics	Value
Age, years (mean ± SD)	67.1 ± 7.9
Male (n, %)	20 (51.3)
Han nationality (n, %)	39 (100.0)
BMI, kg/m^2^ (mean ± SD)	26.6 ± 3.6
Age of hypertension^a^, years (mean ± SD)	11.0 ± 6.3
Complicated other diseases^a^ (*n*, %)	19 (48.7)
Smoke^a^ (*n*, %)	4 (10.3)
Drink^a^ (*n*, %)	5 (12.8)
Exercises^a^ (*n*, %)	4 (10.3)
Antihypertensive agent use	
Calcium channel blockers (*n*, %)	24 (61.5)
Angiotensin II receptor blocker (*n*, %)	21 (53.8)
Angiotensin-converting enzyme inhibitors (*n*, %)	2 (5.1)
Loop diuretics (*n*, %)	4 (10.3)
Beta-receptor blockers (*n*, %)	5 (12.8)
Antiglycemic agent use (*n*, %)	14 (35.9)
Statin use (*n*, %)	1 (2.6)
Visit DBP, mmHg (mean ± SD)	72.6 ± 8.2
Morning SBP, mmHg (mean ± SD)	136.4 ± 18.7
Morning DBP, mmHg (mean ± SD)	76.3 ± 8.7
Night SBP, mmHg (mean ± SD)	134.7 ± 18.1
Night DBP, mmHg (mean ± SD)	74.3 ± 9.7

^a^Age of hypertension: the time since the patient was diagnosed with hypertension by the hospital. Complicated other diseases: complicated other diseases include stroke, coronary heart disease and diabetes mellitus. Smoke: continuing to smoke for more than one year. Drink: drinking more than once a week on average. Exercises: exercise more than three times a week, and not less than 30 min each time.

### Urine test result

After intervention, the 24-h urinary sodium value was 138.05 ± 57.12 mmol/L lower than the baseline level 151.86 ± 55.93 mmol/L, which decreased by13.82 mmol/L (95% CI: − 8.62 to 36.26) with no significant difference (t = 1.246, *P* = 0.220). After intervention, the urine potassium value increased to 61.01 ± 17.26 mmol/L compared with baseline 52.22 ± 19.30 mmol/L, (t = − 2.411, *P* = 0.021), which increased by 8.79 mmol/L (95% CI:1.41 ~ 16.16). The ratio of sodium to potassium was 2.39 (1.44,3.28), which was lower than baseline 3.05 (1.95,4.36) (*P* < 0.001). Among them, the number of patients whose daily NaCl intake < 6 g increased from 8 (20.5%) to 28 (71.8%) at baseline (χ^2^ = 20.635, *P* < 0.001). The 24-h urine microalbumin increased from baseline, *P* < 0.001 (Table 2).

**Table 2.** 24-h urine test results (n = 39).

Observation index	Basline	After intervention	t/T^b^	*P*
Sodium (mmol/24 h)	151.86 ± 55.93	138.05 ± 57.12	1.246	0.220
Potassium (mmol/24 h)	52.22 ± 19.30	61.01 ± 17.26	− 2.411	0.021
Na/K value^a^	3.05 (1.95,4.36)	2.39 (1.44,3.28)	− 3.265	< 0.001
Creatinine (umol/24 h)	12,111.94 ± 3643.72	12,646.85 ± 3410.74	− 1.032	0.309
Microalbumin^a^ (mg/L)	2.00 (1.20,4.10)	2.30 (1.70,5.20)	− 2.727	< 0.001
Calcium (mmol/L)	5.41 ± 2.40	5.81 ± 3.23	− 0.962	0.342
Magnesium (mmol/L)	4.35 ± 1.68	4.77 ± 1.64	− 1.839	0.074

^a^Non-positive distribution data are expressed in median and quartiles, and Wilcoxon Signed Rank Test was used.

^b^t for the statistic of the Paired T Test, and T for the statistic of the Wilcoxon Signed Rank Test.

### Comparison of VBP and HBP

Analysis showed that the difference between the three kinds of blood pressure was statistically significant (SBP: F = 13.220, *P* < 0.001, DBP: F = 24.729, *P* < 0.001). The SBP (− 5.1 mmHg, 95% CI: − 7.1 to − 3.2, *P* < 0.001 Vs − 2.4 mmHg, 95% CI: − 4.4 to − 0.498, *P* = 0.014) and DBP (− 4.8 mmHg, 95% CI: − 6.2 to − 3.5, *P* < 0.001 Vs − 2.0 mmHg, 95% CI: − 3.3 to − 0.6, *P* = 0.004) of VBP were lower than morning HBP and night HBP.

Correlation analysis showed that visit BP was good correlated with morning BP (SBP: r = 0.692, *P* < 0.001, DBP: r = 0.789, *P* < 0.001) and night BP (SBP: r = 0.571, *P* < 0.001, DBP: r = 0.738, *P* < 0.001).

### Changes of blood pressure

The results of mixed linear model analysis showed that patients' visit SBP (− 11.4 mmHg, 95% CI: − 17.0 to − 5.7, *P* < 0.001), morning home SBP (− 10.0 mmHg, 95% CI: − 16.4 to − 3.6, *P* = 0.003) and night home SBP (− 10.2 mmHg, 95% CI: − 15.8 to − 4.6, *P* = 0.001) decreased significantly, after intervention. Besides, visit DBP (− 3.7 mmHg, 95% CI: − 6.7 to − 0.7, *P* = 0.017) and night home DBP (− 4.0 mmHg, 95% CI: − 7.0 to − 1.0, *P* = 0.010) decreased significantly, but the decrease in morning home DBP was not statistically significant. And weekly blood pressure changes are shown in Tables 3 and 4. Besides, we found that VBP decreased during the first week of intervention, while HBP decreased significantly in 2 to 3 weeks.

**Table 3:** Changes in morning SBP from baseline during intervention, mean (95% CI)*.

Week	SBP of VBP		SBP am of HBP		SBP pm of HBP	
	Statistics	P*	Statistics	P*	Statistics	P*
1	− 10.0 (− 15.9, − 4.1)	0.001	− 3.1 (− 9.9, 3.7)	0.360	− 4.8 (− 10.6, 1.1)	0.106
2	− 12.0 (− 17.5, − 6.4)	< 0.001	− 6.2 (− 12.6, 0.2)	0.056	− 7.8 (− 13.3, − 2.3)	0.006
3	− 10.9 (− 16.4, − 5.5)	< 0.001	− 7.6 (− 14.1, − 1.2)	0.021	− 8.6 (− 14.4, − 2.8)	0.004
4	− 13.6 (− 19.9, − 7.3)	< 0.001	− 8.9 (− 15.3, − 2.5)	0.007	− 9.3 (− 14.8, − 3.8)	0.001
5	− 14.4 (− 20.0, − 8.8)	< 0.001	− 9.9 (− 16.2, − 3.6)	0.003	− 11.0 (− 16.5, − 5.4)	< 0.001
6	− 14.0 (− 19.5, − 8.5)	< 0.001	− 8.7 (− 15.1, − 2.4)	0.008	− 10.1 (− 15.6, − 4.6)	0.001
7	− 15.0 (− 20.9, − 9.0)	< 0.001	− 11.0 (− 17.2, − 4.7)	0.001	− 11.9 (− 17.4, − 6.4)	< 0.001
8	− 11.4 (− 17.0, − 5.7)	< 0.001	− 10.0 (− 16.4, − 3.6)	0.003	− 10.2 (− 15.8, − 4.6)	0.001

*Adjusted for sex, age, body mass index , drinking, hypoglycemic agents, antihypertensive drug category and reduction of antihypertensive drugs in linear mixed model.

**Table 4:** Changes in morning DBP from baseline during intervention, mean (95% CI)*.

Week	DBP of VBP		DBP am of HBP		DBP pm of HBP	
	Statistics	P*	Statistics	P*	Statistics	P*
1	− 2.5 (− 5.9, 0.8)	0.134	− 0.6 (− 3.6, 2.4)	0.684	− 1.9 (− 4.9, 1.1)	0.218
2	− 4.0 (− 7.0, − 1.0)	0.009	− 1.7 (− 4.7, 1.2)	0.250	− 3.0 (− 5.9, − 0.1)	0.044
3	− 3.6 (− 6.7, − 0.4)	0.026	− 2.0 (− 5.1, 1.1)	0.202	− 2.7 (− 5.6, 0.1)	0.062
4	− 4.6 (− 7.7, − 1.6)	0.003	− 2.2 (− 5.3, 0.9)	0.157	− 3.3 (− 6.2, − 0.5)	0.023
5	− 4.2 (− 7.0, − 1.3)	0.005	− 3.6 (− 6.4, − 0.8)	0.013	− 3.9 (− 6.8, − 1.0)	0.010
6	− 4.3 (− 7.3, − 1.4)	0.005	− 2.8 (− 5.8, 0.1)	0.061	− 3.4 (− 6.3, − 0.5)	0.021
7	− 3.7 (− 6.9, − 0.5)	0.024	− 3.3 (− 6.1, − 0.4)	0.027	− 4.2 (− 7.0, − 1.4)	0.004
8	− 3.7 (− 6.7, − 0.7)	0.017	− 2.8 (− 5.8, 0.3)	0.072	− 4.0 (− 7.0, − 1.0)	0.010

* Adjusted for sex, age, body mass index , drinking, hypoglycemic agents, antihypertensive drug category and reduction of antihypertensive drugs in linear mixed model.

### Safety

No serious adverse events occurred during the intervention. Other adverse events included: 1 weeks after intervention, 1 patient had constipation. At the third week of intervention, 2 patients developed fatigue, 2 patients developed dizziness or headache, and 1 patient developed itching in the neck, back and abdomen.

## Discussion

The results of this study showed that both the patient's visit SBP and home SBP were reduced by about 10 mmHg, which is more than the results of previous researches (7.52 mmHg)^5^. Besides, it seems that 18% sodium substitute salt not only has a stronger antihypertensive effect but can also lower the blood pressure more rapidly. The results of this study showed that patients had a significant decrease in VBP at the 1st week and HBP at around the 3rd week after using 18% substitute salt, unlike previous studies^24,29,30^. Two studies showed that after restricted-salt intervention in hypertensive patients, blood pressure was significantly reduced in the 4th week^29,30^. And in a prospective cohort study of Hu et al., a substitute salt of 65% sodium chloride was used in a hypertensive patient population, and the patients’ blood pressure was significantly reduced after 18 months of intervention^24^. A better and faster antihypertensive effect may be due to the fact that the 18% sodium substitute salt is rich in potassium and extremely low in sodium. Of course, we cannot ignore the influence of other factors, such as seasonal temperature changes, baseline BP, age, gender and the salt-sensitivity of patients^24,31^. Besides, inevitable health education and salt compliance for patients may also cause interference.

The study on substitute salt began in the 80s of last century. In 1984, Karppanen et al.^32^ found that the substitution of 35–55% potassium and magnesium for sodium ions in traditional salt did not cause excessive poisoning of potassium and magnesium ions. As early as the 1990s, domestic animal toxicity experiments found that LD_50_ in low-sodium group was higher than that in salt group, indicating that low sodium salt was less toxic than that in salt group, and had no accumulation effect in animals^33^. It can be seen that the traditional substitute salt is relatively safe. The substitute salt used in this study is different from the traditional ones due to its sodium and potassium content. From the baseline urine test results, the intake of sodium chloride is about 9 g. We assume that the patient's sodium intake is all from the salt, for ordinary substitute salts containing about 70% sodium chloride, and the daily per capita intake of sodium chloride could be reduced by about 2.7 g. However, the use of substitute salt with only 18% NaCl in this study reduced the daily per capita intake of NaCl by about 7.4 g. Therefore, a substantial reduction of sodium is likely to lead to hypertension patients, especially salt-sensitive patients taking antihypertensive drugs at the same time appear to reduce blood pressure too quickly and some symptoms similar to hypotension. In this intervention, there were individual patients with fatigue, dizziness and other symptoms may be related to this. In addition, the results of this study showed that the patient's 24-h urinary microalbumin was significantly higher than before intervention. 24-h urinary microalbumin (UMA) quantification is the main basis for early clinical diagnosis of nephropathy, 24 h UMA 30 to < 300 mg/24 h is the gold standard for diagnosing early nephropathy^34^. However, after the intervention in this study, the average 24-h urine microalbumin of patients only increased from 2.0 mg/24 h at baseline to 2.3 mg/24 h. The increase was limited, and it is far from reaching the standard for renal impairment. In addition, during our intervention, a male patient found a kidney-changing stone in the hospital, and his urine microalbumin increased significantly. At the same time, due to the small sample size, the patient may have a greater impact on the overall outcome. Of course, we still cannot rule out that this is related to the use of substitute salt.

Previous studies on the comparison between office blood pressure (OBP) and home blood pressure have been conducted, but there was little comparison between the VBP and HBP. Most of the them have shown that the OBP is higher than the HBP^35–37^. In our study, the mean of HBP was higher than the VBP, which was contrary to most studies. It can be explained for the following reasons. First of all, although the visit blood pressure and the office blood pressure were measured by the medical staff, the measurement locations were different. Since the VBP was measured at the patient's home, the patient was in a more relaxed environment and the measured blood pressure may be lower. Al-Karkhi et al.^37^ found that even if hypertensive patients were self-tested at the office, self-test blood pressure was still higher than the self-test blood pressure at home, indicating that the location still has an impact on the patient's blood pressure measurement. What’s more, in the study by Hisao Mori et al.^38^, among sustained hypertensive patients, the mean HBP was lower than OBP by about 5 mmHg, but among normotensive subjects, the controlled hypertensive patients and the masked hypertensive patients, the HBP was higher than the OBP. And other study had similar results^39^. And our study was a phase I clinical trial with limited sample size and a high probability of sample bias, which might lead to the majority of patients in our study who were controlled hypertensive patients or had masked hypertension. Last but not least, since most hypertensive patients take antihypertensive drugs immediately after getting up in the morning, most of the patients have already taken antihypertensive drugs when the followers went to the patients' homes to measure blood pressure, resulting in low blood pressure.

The results of this study show that HBP had a good correlation with VBP, which is similar to the results of Jardim et al.^40^ in adolescents. In addition, the results of Evdokimov et al.^41^ showed that after the intervention, the VBP (from 160.8 ± 8.8/92.6 ± 7.4 mmHg to 125.9 ± 7.9/77.8 ± 5.0 mmHg) decreased more than the HBP (from 147.0 ± 13.3/85.6 ± 7.2 to 127.5 ± 8.3/78,9 ± 5.6 mmHg), and our study seems to have this phenomenon, but we don't clear that whether the difference is statistically significant. What’s more, we observed that VBP seems to drop faster than HBP. And VBP showed a significant decrease in the first week of intervention, while HBP dropped in the second and third weeks, and it can be seen that the decline in HBP seems to be more moderate. This may be related to the instability of VBP as a point sample or related to the difference in blood pressure before and after medication.

## Conclusion

The 18% sodium content of the substitute salt can significantly reduce the patient's VBP and HBP. Although there was a significant difference in the values between VBP and HBP due to the medication, the correlation between them was good, and it does not differ much in the reflection of the intervention effect.
